# Supplementary material for: Are carbon emissions trading and green financial instruments synergistic? -Comprehensive quantitative research based on content analysis
Source: PLoS One. 2024 Mar 7;19(3):e0298601. doi: 10.1371/journal.pone.0298601 (PMC10919601; doi:10.1371/journal.pone.0298601)
Supplement: S1 Data — (ZIP) [file pone.0298601.s001.zip › Data/地级市Z_F工作报告.zip!/┴╔─■/╡ñ╢1⁄2╩╨/└┤╫╘╣1⁄2╓┌║┼í╛┬φ┐╦╩2╛▌═°í┐_2011.docx]

各位代表：

　　我代表市人民政府向大会作政府工作报告，请予审议，并请市政协委员和各位列席人员提出意见。

　　一、“十一五”工作回顾和2010年经济社会发展基本情况

　　“十一五”时期，在省委、省政府和市委的正确领导下，在市人大、市政协的监督支持下，市政府组织和带领全市人民抢抓东北老工业基地全面振兴、国家扩大内需和辽宁沿海经济带上升为国家战略的重大机遇，克服金融危机的不利影响，树立沿江沿海全面开发的雄心壮志，坚定信心，创新思路，锐意进取，真抓实干，超额完成了“十一五”计划的主要任务，经济社会进入新的发展时期。

　　——各项目标圆满完成，主要指标实现翻番。预计2010年实现地区生产总值735亿元，比上年增长17.1%，比2005年增长1.1倍，年均增长16.4%，比“十五”时期提高3.7个百分点;规模以上工业增加值240亿元，比上年增长25%，比2005年增长1.9倍，年均增长24%，比“十五”时期提高4.4个百分点;全社会固定资产投资完成800亿元，比上年增长52.4%，五年累计完成2038亿元，是“十五”时期的5.3倍，年均增长43%;地方财政一般预算收入实际完成80.2亿元，比上年增长38%(新体制可比口径)，比2005年增长2.7倍，年均增长30.2%;实际利用外商直接投资7亿美元，比上年增长40%，五年累计17.7亿美元，是“十五”时期的13.1倍，年均增长60%;外贸出口总值17亿美元，比上年增长17.3%，按可比口径比2005年增长1.03倍，年均增长15.2%;引进内资项目到位资金505亿元，比上年增长110%，三年累计到位资金866亿元;全社会消费品零售总额275.6亿元，比上年增长18.6%，比2005年增长1.3倍，年均增长18%;城镇居民人均可支配收入14536元，农民人均纯收入8295元，比上年分别增长13.3%和13.7%，年均分别增长16.1%和14.7%。

　　——丹东新区迅速崛起，城市空间得以扩展。全面开发建设以新区为核心的沿江沿海经济带，丹东经济实现由沿江向沿海发展的快速转移，城市发展空间已经拉开。新区率先启动，丹东人多年的梦想得以实现，仅两年多的时间，在一片荒地之上建立起丹东新的行政经济文化中心雏形，累计开发土地面积22平方公里，基础设施及配套工程基本完成，开工建设项目412个。其中，市民服务中心、丹东二中新校区等城市功能项目投入使用，SK丹东保税物流中心、新城区商业街及新加坡城等商住项目已经竣工，帕斯特谷物大豆加工、曙光SUV乘用车等工业项目投产见效，文化中心、体育中心、本钢不锈钢等项目正在加紧建设。仪器仪表产业基地仅一年半的时间，45万平方米的标准厂房和研发检测中心、物流配送中心全部投入使用，签约仪器仪表企业320户，入驻企业210户，被誉为“丹东速度”。环保产业园区着力打造新兴产业，开工建设项目20个，总投资46.5亿元。大孤山经济区的海洋红港区建设已经启动，东港经济区的辽宁再生资源产业园获得国家“圈区管理”审批，前阳经济区的佰山木业等项目落地实施，通远堡、金山、汤池、五龙背等重点区域成为全地区开发开放的新增长点。丹东东港同城化实现全方位推进。

　　——重大项目陆续启动，投资拉动成效显著。五年累计开工建设千万元以上项目1669个，投资总额2890亿元;竣工项目1057个，投资总额1625亿元，项目建设促进固定资产投资快速增长，特别是2008年以来连续三年增长50%以上。先后开工建设丹通、丹海两条高速公路和东北东部铁路、沈丹客专、丹大快铁三条铁路，改扩建丹东机场，新建中朝鸭绿江大桥，多年企盼的重大项目得以实施。丹东港建设五年累计投资181亿元，吞吐量达到5500万吨，为打造东北东部新的出海通道奠定了基础。蒲石河抽水蓄能电站、三湾水利枢纽及输水工程、金山热电等重大电力能源项目相继启动并快速建设，丹东首座500千伏变电站顺利投入运行，使我市向辽宁能源大市迈进。首钢翁泉沟硼铁资源综合利用、盛大恒通高新管业等工业项目，成达肉食鸡加工等农业项目，东北亚国际商贸城等商贸物流项目及北国之春度假村等旅游项目正在积极建设，有的已经竣工投产。(下转第二版)

　　——工业实力明显增强，产业集群初步形成。规模工业增加值增速连续几年位居全省前列，三次产业比重由2005年的17.5:40.6:41.9调整为13.6:49.9:36.5，工业的拉动支撑作用逐步呈现。工业投入五年累计完成930亿元，是"十五"时期的12.3倍，年均增长62.4%;实施投资500万元以上工业项目2000个，是"十五"时期的2倍。主业收入亿元以上企业达到193户，是2005年的近4倍，曙光集团成为我市第一个百亿元企业。工业园区的快速发展带动了产业集聚，我市已经建设和正在建设工业园区40个，形成主业收入超10亿元产业集群11个，其中汽车及汽车零部件和农产品加工两大产业集群规模超过百亿元。科技创新步伐加快，企业技术研发体系建设形成国家、省、市联创格局，高新技术产业增加值五年增长6倍。国企改革取得重大突破，企业活力不断增强，职工权益得到妥善保障。

　　--特色农业初具规模，县域经济提速升位。农民人均纯收入连续五年实现两位数增长。农产品出口创汇达到6亿美元，稳居全省第二位。粮食生产稳步发展，2010年虽遭遇洪涝灾害，仍然高于平年水平。优质稻米、食用菌、小浆果等一批特色产业规模跻身全省乃至全国前列，我市成为全国最大的板栗生产、加工、出口基地，东港获得全国优质草莓生产基地命名。渔业养殖和畜牧业养殖快速发展，设施农业规模是"十五"期末的2倍，亿元以上农产品加工企业达到25户，是"十五"期末的3倍，伏季休渔、增殖放流取得显著成效。无公害产地认定和产品认证由零增加到120个。2009年夺得省农建"大禹杯"。农田水利建设五年累计投资17.9亿元，解决了44.9万农村人口的饮水安全问题。林权制度主体改革全面完成。东港市率先进入全国百强县并排在第83位。县域产业集群发展迅速，东港的再生资源、机械加工、纺织服装、食品加工，凤城硼铁资源综合利用和宽甸硼镁化工等产业集群已具备超百亿元规模的实力。

　　--商贸旅游日渐繁荣，城区经济蓄积后劲。乐购、华润万家、大商新玛特、沃尔玛等一批国际国内知名商业集团相继入驻丹东。借助国家拉动内需等政策，在全地区实施了"家电下乡"、"万村千乡市场工程"等城乡流通网络建设，累计建设标准连锁农家店906家，新建和改建配送中心10个，80多万农民直接受益。推出以鸭绿江为核心的旅游品牌，虎山长城等风景名胜区环境设施极大改善。2009年，我市成为全国首批边境旅游异地办证试点城市，丹东鸭绿江国际旅游节实现经贸和旅游的高度融合，全面展示了丹东对外开放的成果和水平，成为老百姓自己的节日。旅游客源市场延伸到长三角、珠三角和港台等地区。城区经济通过下放管理权限和建设服务业集聚区、工业园区，实力显著增强。振安区率先启动行政中心外迁，带动城区发展空间快速拓展。

　　--招商引资拉动强劲，开放水平不断提高。大力开展仪器仪表、环保等产业的主题招商、全员招商，引进大量资金及重大项目。外资引进大幅增长，特别是2008年以来，实际利用外资三年翻了两番多。世界500强企业韩国SK、香港华润、美国哈曼相继落户丹东。口岸建设极大促进了外经外贸发展，我市率先获准进入国家边境贸易人民币结算试点。承办大型活动能力和接待水平显著提升，连续三年成功举办了辽宁"台湾周"。携手大连及东北东部11市(州)，初步构建了东北东部(12+1)区域合作新框架，鸭绿江经济合作先行区第一个先导项目--通丹"陆港"正式开工建设。

　　--公共财政保障有力，金融信贷增速加快。财政一般预算收入连续五年保持了30%以上的增速，占经济总量的比重五年上升了5.3个百分点。财政支出始终突出民生重点，五年间共向社保、医疗、就业、教育等民生领域投入资金301亿元，支出增量80%以上用于民生保障。2010年，预计全市各项存款余额940亿元，是"十五"期末的2倍;贷款规模达到478亿元，贷款增量由年度平均20亿元提高到101.3亿元。2010年锦州银行、大连银行登陆丹东，打破了丹东地区13年无银行机构进驻的局面，形成13家银行机构、22家保险公司、5家村镇银行及15家小额贷款公司为主体的金融市场体系。成功发行15亿元市政建设项目债券，加速推进企业上市自主融资，担保业快速发展，基金和中小企业集合票据融资实现突破。

　　--城市建设亮点增多，民生实事惠及百姓。投资近20亿元用于城市出口、城市道路、内河治理、立面改造等工程，三条快速路、多条城市主干线全面贯通。实施城市自来水管网改造工程，引入城市燃气新气源。5座污水处理厂、生活垃圾卫生填埋场投入使用。新建城镇住宅1100万平方米，城市人均住房建筑面积达到23平方米。实施集中连片棚户区改造，安置居民近2万户。城市管理水平进一步提高，城市面貌焕然一新。兴办惠民实事43件，涉及群众生活的问题得到逐步解决，残疾人等特殊群体的合法权益得到有效保障。五年累计实现就业31万人次，城镇登记失业率从"十五"末期的5.5%下降到4.07%以内。各项社会保险扩面和基金征缴力度不断加大，保障水平逐步提高。城市低保户月救助标准、农村低保户年救助标准五年均提高一倍多。新建配建廉租房、经济适用住房2000余套，为农村困难户建房8819户。投资6亿元为183个村通油路1385公里，2009年提前一年实现了村村通油路目标。

　　--社会事业全面发展，和谐丹东积极构建。新一轮义务教育普九工作加快推进，基本普及高中阶段教育。移地新建丹东二中、四中、职业中专等项目，改造中小学危房27.6万平方米，教育设施得到较大改善。基本建立起覆盖城乡居民的医疗保障体系，市、县、乡医疗基础设施建设取得重大进展，卫生救治能力和人均预期寿命明显提高。低生育水平持续稳定。第六次人口普查顺利推进。全民健身活动普及提高，群众体育和竞技体育健康发展。完成了广播电视"村村通"工程和抗美援朝纪念馆等公共文化基础设施建设。引智工作积极推进，五年共引进博士及国外经济和文教专家440多人次，引进海外研发团队项目15项。精神文明建设日益深入，军民军政团结、双拥模范城和爱民固边模范市创建成果进一步巩固。外事及侨务管理日趋规范。"平等、团结、互助、和谐"的社会主义民族关系得到巩固发展，宗教界"双文明"争创活动成果显著。坚持向人大及其常委会报告工作和向人民政协通报情况制度，认真听取各民主党派、工商联、无党派人士和各人民团体的意见。五年共办理人大代表建议1124件、政协提案1280件，办结率100%，满意率和基本满意率100%。档案信息化建设推进顺利，地方志修编工作取得成效。"五五"普法顺利完成，行政审批制度改革进一步深化，行政效能和软环境建设不断加强。积极创建平安丹东，全市信访总量明显下降，社会治安秩序明显好转，圆满完成北京奥运会、国庆60周年等重大活动安保工作，市巡警五大队被国务院授予"执法为民巡警大队"光荣称号。防震减灾、气象服务、城市总体防护能力全面提升。采取有效措施调控物价，保持了市场稳定。安全生产工作进一步加强，食品药品等市场秩序进一步规范，人民群众切身利益得到有效保障。环境保护已见成效，"十一五"期末，全市化学需氧量和二氧化硫排放总量分别比2005年下降13%和12%。

　　--抗洪抢险取得胜利，灾后重建全面展开。2010年，我市遭遇建国以来第二大暴雨洪水的袭击，持续时间之长、降雨强度之大、洪水袭击之猛、抗灾救灾之难，均为历史罕见。面对严重的洪涝灾害，全市党政军民在省委、省政府的正确领导和大力支持下，万众一心，众志成城，不畏艰险，敢于牺牲，夺取了抗洪抢险救灾的重大胜利。由于准备充分，调度科学，预测准确，群众转移及时，全市没有发生重大伤亡;由于部署周密，预案完备，多方配合，应急工作细致到位，灾后没有出现重大疫情;由于宣传深入，抢险有效，救灾有方，生产自救措施落实有力，人民群众生活没有受到重大影响;由于团结一心，奋力拼搏，在最短时间内转入经济建设中，丹东经济依然保持快速发展态势。目前，灾后重建工作全面展开，救助措施迅速落实，各项水毁修复工程积极推进，特别是房屋重建工作在灾后立即启动，现已近尾声，1300户重建房屋中已有1214户达到入住条件，确保了受灾群众安全、温暖过冬。

　　各位代表，回顾过去五年的发展历程，我们深感成绩来之不易。五年来，我们以科学发展观为统领，以和谐发展、跨越发展为目标，始终坚持把市委的领导作为全市工作的统一步调，始终坚持把人大和政协的监督作为推动政府工作的重要保证，始终坚持把依靠和凝聚全市人民力量作为实现跨越发展的强大动力，始终坚持把狠抓落实作为完成各项任务的有效举措，始终坚持把人民群众利益作为开展工作的出发点和落脚点。这是过去五年我们取得工作成绩的原因所在，也是未来再接再厉、再创佳绩的重要经验和法宝。在此，我谨代表市政府，向全市广大工人、农民、知识分子和各级干部，向驻丹人民解放军指战员、武警官兵、公安干警和中省直驻丹单位，向关心和支持政府工作的各民主党派、工商联、无党派人士、各人民团体和社会各界人士，表示衷心的感谢，并致以崇高的敬意!

　　回顾过去，成绩固然令人欣慰，但我们也清醒地看到，我市经济社会发展还存在着一些亟待解决的困难和问题，突出表现在：一是经济总量偏小，结构不够合理。二是有竞争力的大企业、大集团较少，后续牵动性大项目不多。三是资金、土地等要素供需矛盾日渐突出。四是涉及群众切身利益的一些问题还没有得到根本解决，社会保障、就业形势仍然严峻。五是政府管理和服务水平有待进一步提高。对此，我们将采取切实有效的措施加以解决。

　　二、"十二五"时期发展思路和奋斗目标

　　"十二五"时期，是深入贯彻落实科学发展观、加快转变经济发展方式、构建社会主义和谐社会的重要时期，是丹东沿江沿海经济蓄势而发、书写辉煌的有利时期，更是实现丹东经济跨越发展、社会和谐平安的关键时期。未来五年，国际国内环境中的风险与机遇并存，省内发展中的竞争与共促并存，丹东自身发展中的压力与潜力并存。我们必须在矛盾的交织中，寻找和抓住机遇，咬住跨越目标不放松，迎难而上，乘势而起，实现丹东的新跨越。在全面分析形势，认真听取各方意见的基础上，我们编制了《丹东市国民经济和社会发展第十二个五年规划纲要(草案)》，确立了今后五年全市经济和社会发展的总体思路和奋斗目标。

　　"十二五"期间，全市经济和社会发展的指导思想是：高举中国特色社会主义伟大旗帜，以邓小平理论和"三个代表"重要思想为指导，深入贯彻落实科学发展观，全面落实市委十届十一次全会精神，紧紧抓住辽宁沿海经济带上升为国家战略的机遇，以科学发展、创新发展、和谐发展为主题，以加快转变经济发展方式为主线，坚持增量带动结构优化、创新促进产业升级、发展保障民生改善，显著增强综合经济实力，显著提高人民生活质量和水平，把丹东建设成为"文明、生态、繁荣、富裕"的宜居佳地，努力构建大气秀美的新丹东。

　　"十二五"时期，我市经济社会的主要奋斗目标是：

　　一是综合经济实力整体突破。地区生产总值年均增长20%，到2015年，达到1800亿元，比"十一五"期末再翻一番以上，力争突破2000亿元;人均地区生产总值超过1万美元;地方财政一般预算收入年均增长28%，达到275亿元，力争突破300亿元;全社会固定资产投资年均增长30%，五年累计投资9000亿元，力争突破10000亿元。县域经济实力显著增强，东港市进入全国五十强，凤城市进入全国百强，宽甸县进入全省十强。

　　二是开放水平全面提升。实际利用外商直接投资年均增长37%，累计突破100亿美元;引进内资项目到位资金年均增长36%，累计突破5000亿元;外贸出口年均增长20%，到2015年，达到42亿美元。以中朝鸭绿江大桥开工建设为契机，以东北东部铁路、丹东新机场等基础设施工程投入使用为依托，引进世界500强企业5户以上，打造辽宁对外开放的新高地。丹通和丹海高速公路、东北东部铁路、沈丹客专、丹大快铁全部通车，港口吞吐量达到1.5亿吨，开工建设新机场，形成完备的交通基础设施体系，成为东北东部新的出海通道和东北亚区域中心城市。对朝贸易出口总值年均增长20%，达到15亿美元，成为全国对朝鲜半岛开放的桥头堡。以四大经济区为代表的沿江沿海经济率先发展、快速崛起。

　　三是经济结构更趋优化。工业经济总量持续扩张，汽车及汽车零部件、钢铁等材料、电子信息、农产品加工四个支柱产业壮大做强，新兴产业和高新技术产业比重大幅提高，形成特色鲜明的主导产业，三次产业比例调整为7：51:42。

　　四是城市化进程明显加快。拓展城市发展空间，以新区、大孤山两大新城建设为牵动，实现城市发展中心向西转移。加快城镇建设，引导人口向县城和中心城镇集中，用工业化推动城镇化，用城镇化带动农业现代化，构建辐射全域的城镇化。全地区常住人口达到300万人，地区城镇化率达到70%。

　　五是生态环境和谐优美。大力倡导低碳生活，加大节能降耗力度，积极发展循环经济，建成区绿化覆盖率争取达到40%，森林覆盖率达到67.5%。开展"四城同创"，即全国卫生城、全国环保模范城、国家园林城市、全国文明城。

　　六是人民生活水平大幅提高。社会保障体系进一步完善，城乡救助体系实现全覆盖。就业更加充分，城镇登记失业率控制在4%以内。人民生活明显改善，到2015年，城镇居民人均可支配收入达到全省平均水平，农民人均纯收入达到全省先进水平。

　　为实现上述目标，今后五年我们将全力做好以下工作：一是以四大经济区建设为重点，形成丹东经济发展核心区域;二是以对外开放为主战略，强化物流集散、进出口加工和国际商务会展等功能;三是以"五项工程"为主线，实现工业经济总量翻两番;四是以特色农业产业基地建设为龙头，促进农业产业化进程;五是以改造提升消费性服务业为主导，促使现代服务业成为地区经济的重要支撑;六是以资源优势为依托，尽快提升县区经济整体实力;七是以城市拓展和基础设施建设为突破口，早日建成东北东部区域性交通中心;八是以构建和谐社会为目标，建设保障有力、平安有序、生态优良的现代都市。

　　三、2011年工作目标和主要任务

　　市委、市政府确定今年的工作主题为"项目建设年"，全市经济和社会发展主要预期目标是：地区生产总值增长17%，规模工业增加值增长20%，全社会固定资产投资增长25%，地方财政一般预算收入增长25%，实际利用外商直接投资增长42%，外贸出口总值增长18%，引进内资项目到位资金增长50%，全社会消费品零售总额增长18%，城镇居民人均可支配收入增长13%，农民人均纯收入增长12%，城镇登记失业率、居民消费价格总水平控制在全省平均水平以内。为实现上述目标，今年要全力抓好以下重点工作：

　　(一)以重大项目建设为带动，实现投资规模大幅增长

　　全年计划实施亿元以上项目328个，投资总额2846亿元，完成投资680亿元，力争引进超百亿元以上重大项目。抓紧新开工一批项目。深入实施市级领导包扶重大项目制度，加强调度，切实落实项目开工责任主体等事项，加快项目要件的审批，为项目开工创造条件。抓紧竣工一批项目。建立高效快捷的推进机制，抓好项目的跟踪、推进和落实，抓重点、攻难点，抓节点、求突破，确保接转项目早见形象、早日竣工、早出效益。抓紧引进一批项目。坚持"走出去，请进来"，突出我市政策、资源、区位等方面的优势，加强与央企的合作、与世界500强企业的对接，引进一批具有强劲拉动力和核心竞争力的大项目，提高全市经济运行质量和效益。抓紧推进一批项目。紧紧抓住国家产业结构调整的有利时机，紧跟国家项目投资的导向，加强与国家、省的沟通、衔接，争取有更多项目纳入国家、省计划之中。

　　(二)以江海一线率先发展为突破，加快四大经济区开发建设

　　加快新区开发开放。全方位、高层次、多渠道推介新区，做精基础设施、标志性建筑及各种配套工程，提升新区城市品位。以安民山为核心，继续拓展新区开发区域，启动现代服务业等5个新片区建设，实现行政中心等城市功能项目开工建设，新区开发面积达到30平方公里。确保景观工程竣工，仪器仪表学院、职业中专、九年义务教育学校投入使用，新区快速向新城转变。仪器仪表产业基地新建标准厂房50万平方米，投产企业300户; 环保产业园新开工和续建超亿元项目25个;装备制造园起步区、不锈钢产业园项目摆满;日本、台湾、手表等工业园区加快企业入驻，现代物流等园区抓紧开工建设。

　　加快大孤山经济区建设步伐。全面做好经济区整体和分项规划，着力发展临海临港产业，形成重大项目集聚的新经济增长区域。快速推进海洋红港区东防波堤、航道试挖槽、3万吨和7万吨级两个通用码头、20万吨级矿石专业码头建设，争取如期完工。开工建设大盘线孤山高速出口至201国道4.7公里拓改项目，畅通港区运输条件。临港重化工、物流、铸造机加工与阀门等园区加紧建设并达到企业入驻标准。大洋河引水工程完成论证上报，争取国家资金支持。

　　加快前阳、东港经济区开发建设。迅速将产业项目、工业园区与新区、大孤山两大经济区连接成线，打造成一条新的产业连接带。前阳经济区推进30平方公里核心区的路网等配套设施建设，新开工一批重点项目。东港经济区再生资源产业规模力争突破百亿元。

　　(三)以五项工程实施为支撑，推进工业集群快速壮大

　　企业提升工程：大力发展百户重点企业，壮大曙光等一批龙头骨干企业，全年新增主业收入超10亿元的企业2户，超20亿元的企业1户，超30亿元的企业3户。

　　项目建设工程：完成工业投入400亿元，力争达到500亿元。重点推进100个超亿元工业项目，突出抓好18个10亿元以上的重大工业项目，加快项目建设和投产。

　　产业集群工程：抓好汽车及汽车零部件、仪器仪表、再生资源综合利用三大主导产业集群，集中建好12个重点工业园区。加快发展无水印染等高新技术产业和新兴产业。

　　企业并购工程：实施5项海外并购项目，争取完成辽宁天赐集团等3个并购项目。

　　节能淘汰工程：严格执行行业准入条件，鼓励发展循环经济，规模以上工业能耗下降4%，淘汰落后产能130万吨。

　　(四)以特色产业拉动为依托，增强县域经济综合实力

　　大幅提升县域实力。东港市全国百强位次前移，凤城市冲击全国百强，宽甸县冲击全省十强。凤城硼铁产业及黄金、宽甸有色金属及石材等特色产业集群全力上规模，带动县域产业集群提升壮大。切实抓好县城规划、园区开发和小城镇建设。

　　大力发展特色农业。稳定粮食生产。加快现代农业集聚区和特色农业产业基地建设，加快优质稻米、精细蔬菜、食用菌、小浆果、板栗、中药材等产业发展。抓好设施农业建设，加快畜牧业、特色水产养殖业发展。规范和发展农民专业合作社，扶持规模龙头企业，建设市级标准化示范基地，推进农业标准化生产。加大农产品品牌创建力度，健全品牌保护机制。深化科技兴农，深入开展农村科技特派员工作。

　　全力抓好"菜篮子"。科学制定"菜篮子"工程发展规划，加大新菜田开发的资金投入，保证菜田的占补平衡。扩大设施蔬菜生产面积，落实设施农业小区建设扶持政策，强化生产技术指导，确保设施蔬菜质量安全。做好产销对接，稳定蔬菜价格。扩大肉蛋奶生产规模。

　　加快灾后重建工作。充分利用今年汛前时间，抢修被毁河道堤防、险工险段，恢复提升防洪标准，基本完成病险水库除险加固，确保度汛安全。抓紧修固灌渠、蓄水、排水工程，保证水田灌溉用水。及时修复水毁农村人畜饮水工程。继续抢修农建工程，确保今年春耕生产。抓紧修复损毁公路、桥梁、电力、通讯等基础设施和农业综合开发项目，稳步提高灾区农业综合生产能力，让受灾群众的生产生活恢复到灾前水平。

　　(五)以现代服务业驱动为重点，提升城区经济发展水平

　　加快发展城区经济。全面完成财政收入"三年倍增"目标。按照科学有序、均衡匹配的原则，完成城市行政区划调整。进一步做好城区管理权限的下放工作，大力推进服务业集聚区和工业园区建设。拓展城市发展空间，加快发展东部、北部经济，逐步把金山、九连城、汤池、五龙背建成各具特色、充满活力的卫星城。

　　加快发展商贸流通业。全力打造东北亚国际商贸城等现代服务业集聚区，以及五龙背温泉之都等特色服务业集聚区。促进房地产业持续健康发展。抓好小城镇商业中心建设，继续开展"家电下乡"、"万村千乡市场工程"以及农产品现代流通网络体系建设等惠及农民的重点工程。

　　加快发展旅游业。推进鸭绿江精品旅游带建设，整体包装推介鸭绿江景区、大鹿岛景区，力争引进资金进行整体开发。抓好赴朝旅游集聚区、凤凰山景区、乡村旅游等重点旅游项目建设，天桥沟旅游综合开发项目实现旅游休闲场所部分开放。合理规划利用温泉资源，提升温泉旅游档次，逐步打造鸭绿江温泉休闲旅游集聚区。整合特色景点旅游资源，做好整体包装宣传。进一步巩固和拓展旅游客源市场，加强东北东部、环渤海、华北地区旅游合作。

　　(六)以中朝鸭绿江大桥建设为契机，拓展开放全新领域

　　内资外资同步增长。抓好外资项目的落地履约、资金到位率，确保实际利用外商直接投资完成10亿美元。加快中朝鸭绿江大桥建设，积极争取大桥口岸区及物流园区早日开工。举办好与央企、基金公司的对接洽谈会，与港商、台商的重大项目推介会，以及温泉主题招商会、仪器仪表博览会，实现内资外资到位的新增长。

　　外贸外经齐头并进。扩大外贸出口，加快设立境外经济合作区的步伐，争取物流配送、国际贸易、资源产品加工等项目开工建设。积极推进境外投资。尽快完成综合保税区的前期工作，争取获批建设。抓紧规划建设中朝鸭绿江跨境自由贸易区。

　　区域合作实质推进。拓宽东北东部(12+1)区域合作领域并推向深入，加快建设区域合作总部大厦，年底前完成"通丹"陆港建设并投入使用。继续办好鸭绿江国际旅游节和辽宁"台湾周"活动。

　　(七)以破解金融、土地瓶颈为切入点，全力保障建设需求

　　强化财政的杠杆作用。坚持依法征管，努力做大财政体量。优化支出结构，全力保障民生、灾后重建等支出。进一步加强政府性债务的管理，切实防范财政风险。

　　强化金融的支持作用。积极扩大信贷规模，融资总量争取达到200亿元。新增5家以上金融机构。争取2家企业上市。

　　强化土地的保障作用。积极倡导节约集约利用土地，提高土地利用强度。通过拆旧建新、土地整理复垦等方式，全力推进城乡建设用地增减挂钩工作，并积极探索造地新途径，缓解用地紧张难题。规范土地交易行为，实行净地出让。

　　(八)以深化改革为动力，创新管理体制机制

　　深化财政管理体制改革。全面实施新的财政管理体制，统筹兼顾各级政府利益，按照财权和事权相匹配原则，努力促进各级财政收入稳定、协调和快速增长。建立规范的转移支付制度，平衡地区间的财力差异，确保新财政体制平稳运行。

　　深化城区经济管理体制改革。按照"合理划分事权、强化基层工作、明晰责权配置、事权财权对称、落实城区职责"的原则，合理赋予城区更多的经济发展管理权限。凡是能由城区管理的权限，都要因地制宜，落实到位，充分激发城区经济活力。

　　创新城市管理体制。积极推行两级政府三级管理体制，尽快向区、街下放城市管理权限，加强属地管理，切实做到责权利相统一。加大对区街两级环境卫生人员、经费和设施投入，切实解决背街小巷、破损小区的脏乱差问题。

　　创新沿江沿海经济区干部管理体制。进一步下放相关市级管理权限，赋予各经济区更多自主权。全面实行全员聘任和绩效工资制度。公开招聘管理人员和高层次专业人才，为沿江沿海开发开放提供人才、智力保证。

　　(九)以办好惠民实事为抓手，提高人民群众生活质量

　　办好15件惠民实事。1、实施城市内河综合整治工程;2、实施城市老旧建筑节能保温改造工程;3、改造城市区破损路;4、新建新区市民广场;5、新建安民山公园;6、实施同兴镇及金山镇供水工程;7、改造城市燃气储罐，提高供气能力;8、实施城区公厕改造工程;9、建设新老城区连接带沿江景观步道;10、启动建设百公里"山水相连边城大众健身步道";11、实施棚户区改造工程;12、实施新区安居工程;13、建设2.2万平方米、440套廉租房;14、建设202个标准化村卫生室(所);15、修复渔业资源，增殖放流大规格鱼虾苗种6000万尾。

　　完善社会保障体系。继续做好实名制就业、普惠制培训，全年实名制就业5万人。强化养老、失业、医疗、工伤、生育等保险的扩面工作，加大保费的征缴、入库和对有关违规企业的处罚力度，确保离退休养老金按时足额发放。进一步做好城乡低保、残疾人等社会困难群体基本生活保障，更加关注农村困难群众生活，大力推进养老服务体系建设。

　　加强城市建设管理。做好城市总体规划的修编工作。实施三号干线东尧段、二号干线东段等城市道路工程，力争开工建设四号干线。高标准规划建设市政基础设施。抓好城市公园及风景区建设，扩大城市绿化面积。深入开展环境综合整治，实施立面改造及对市区违法违章建筑的拆除。抓好减排和生态环境建设，改善群众生活环境。

　　(十)以社会事业均衡发展为统筹，促进平安丹东和谐稳定

　　全面发展社会各项事业。优化资源配置，加强学前教育工作，大力发展职业教育。广泛开展贫困助学活动。积极落实医改五项任务，推进中心医院改扩建等卫生基础设施建设，提升妇儿卫生保健水平。大力发展文化产业，丰富群众文化、体育活动，认真做好全国、省"十二运"的筹备工作。深入开展民族团结进步创建活动，大力实施"兴边富民"行动，积极引导宗教与社会主义社会相适应。认真抓好全民国防教育，进一步加强民兵预备役和边海防建设，深入开展双拥模范城创建活动。大力抓好人才工作，为各类人才的引进提供政策、资金保障。

　　加快推进精神文明建设。深入开展文明社区、文明行业、文明单位、文明镇村创建活动，推进"四城同创"。加强未成年人思想道德建设。注重发挥新闻舆论的引导和监督作用，倡导文明和谐的社会风尚。抓好诚信建设，提高政府公信力。

　　全力维护社会和谐稳定。开展"六五"普法活动;全面加强信访工作，努力解决群众合理诉求;加强边境地区治安难点、热点问题的整治和国家安全工作;扎实推进治安防控体系建设，开展系列平安创建活动和社会管理创新工作;抓好安全生产，坚决遏制较大安全生产事故的发生;有效防控和应对重大突发事件;稳定市场物价;加强食品药品等安全监管。

　　(十一)以切实转变职能为手段，努力建设勤政高效廉洁政府

　　扎实推进依法行政。规范政府权力运行，完善执法程序。健全科学民主的决策机制，严格执行重大事项集体讨论决定制度，完善公众参与、专家咨询和政府决策相结合的机制。强化国有资产监督管理运营体制，确保国有资产的保值增值。大力推行行政问责制，强化责任追究。自觉接受人大、政协的监督，认真听取民主党派、工商联、无党派人士和各人民团体的意见，问需于民、问政于民、问计于民。

　　努力提高行政效能和服务水平。继续深化行政审批制度改革，提高审批效率。深化效能监察，规范执法行为，促进软环境进一步改善。完善和实施好"绿色通道"、"一站式"服务等项目推进机制，加快重大项目落地。推进电子政务，深化政务公开，推动政府信息公开向基层延伸。

　　进一步转变工作作风。大兴调查研究之风，注重深入基层、深入群众，掌握真实情况，解决具体问题。减少文山会海，严格控制行政支出。加强督查督办，大力倡导明察暗访、现场办公等方式。巩固"落实年"成果，坚持办实事、求实效，集中精力，真抓实干，通过抓落实来推动经济社会加快发展。

　　着力加强廉政建设。大力推进惩防体系建设，认真抓好"小金库"等专项治理，切实从源头防治腐败。完善政府采购、工程招投标制度，扩大政府采购和招标范围。强化审计工作。完善政风行风评议制度，坚决查处和纠正损害群众利益的不正之风。

　　各位代表，未来五年，困难更能考验我们的能力，挑战更能磨炼我们的意志，团结更能凝聚我们的力量，奋斗更能铸就我们的辉煌。我们一定要在市委的坚强领导下，在市人大、市政协的监督支持下，与全市人民一道，万众一心，振奋精神，迎难而上，开拓进取，以优异的成绩向建党九十周年献礼!
